# First Report of 13 Species of *Culicoides* (Diptera: Ceratopogonidae) in Mainland Portugal and Azores by Morphological and Molecular Characterization

**DOI:** 10.1371/journal.pone.0034896

**Published:** 2012-04-19

**Authors:** David W. Ramilo, Suraya Diaz, Isabel Pereira da Fonseca, Jean-Claude Delécolle, Anthony Wilson, José Meireles, Javier Lucientes, Rita Ribeiro, Fernando Boinas

**Affiliations:** 1 Interdisciplinary Centre of Research in Animal Health (CIISA), Veterinary Medicine Faculty, Technical University of Lisbon, Lisbon, Portugal; 2 Laboratory of Entomology, Institute of Parasitology and Tropical Pathology, Faculty of Medicine, Strasbourg, France; 3 Pirbright Laboratory, Institute for Animal Health, Pirbright, Woking, Surrey, United Kingdom; 4 Parasitology and Parasitic Diseases, Department of Animal Pathology (Animal Health), Veterinary Faculty, University of Zaragoza, Zaragoza, Spain; University of Minnesota, United States of America

## Abstract

The genus *Culicoides* (Diptera: Ceratopogonidae) contains important vectors of animal and human diseases, including bluetongue, African horse sickness and filariosis. A major outbreak of bluetongue occurred in mainland Portugal in 2004, forty eight years after the last recorded case. A national Entomological Surveillance Plan was initiated in mainland Portugal, Azores and the Madeira archipelagos in 2005 in order to better understand the disease and facilitate policy decisions. During the survey, the most prevalent *Culicoides* species in mainland Portugal was *C. imicola* (75.3%) and species belonging to the Obsoletus group (6.5%). The latter were the most prevalent in Azores archipelago, accounting for 96.7% of the total species identified. The Obsoletus group was further characterized by multiplex Polymerase Chain Reaction to species level showing that only two species of this group were present: *C. obsoletus sensu strictu* (69.6%) and *C. scoticus* (30.4%). Nine species of *Culicoides* were detected for the first time in mainland Portugal: *C. alazanicus*, *C. bahrainensis*, *C. deltus*, *C. lupicaris*, *C. picturatus*, *C. santonicus*, *C. semimaculatus*, *C. simulator* and *C. subfagineus*. In the Azores, *C. newsteadi* and *C. circumscriptus* were identified for the first time from some islands, and bluetongue vectors belonging to the Obsoletus group (*C. obsoletus* and *C. scoticus*) were found to be widespread.

## Introduction

The genus *Culicoides* (Diptera: Ceratopogonidae) includes 1.316 recognised species [1]. Adult female *Culicoides* are haematophagous and feed on a wide range of hosts including humans, livestock and other mammals, amphibians, and birds. *Culicoides* can transmit pathogens responsible for several diseases with veterinary and public health significance, including bluetongue (BT) in ruminants, African horse sickness (AHS) in equids, epizootic hemorrhagic disease (EHD) in deer, and filarial diseases such as onchocercosis and mansonellosis, which affect various species including humans [2], [3]. Bites may also cause hypersensitivity reactions in equines, a condition known as “sweet itch”. *Culicoides* larval habitats are aquatic or semi-aquatic, and different species utilize habitats with a broad range of salinity and acidity, for example salt marshes and peat bogs. Species of veterinary interest generally feed on livestock and horses, and breed in associated habitats such as leaf litter, rotting manure and organically-enriched mud.

An outbreak of AHS occurred in mainland Portugal in 1989 [4], and BT transmission resumed in mainland Portugal in 2004 [5], [6], 48 years after the last recorded case. This occurred during a period in which bluetongue virus (BTV) activity was increasing across Europe as a whole. *Culicoides*-borne viruses are introduced into new regions through windborne transportation of *Culicoides* or by accidental importation of infected hosts [5], [6]. Climate change was thought to have facilitated spread into Europe [7], [8], which may have allowed species such as *C. imicola*, which prefer hot, dry conditions, to expand their range in southern Europe as well as increasing vector density, their seasonal activity period and/or their susceptibility to infection with viruses. As a result of increasing global trade [9] and the proximity of the Iberian Peninsula to the North of Africa, the likelihood of new *Culicoides*-borne diseases being introduced into Portugal is likely to remain high for the foreseeable future.

In recognition of this, the Portuguese authorities established a National Entomological Surveillance Program (ESP) in 2005. The objective was to collect and identify *Culicoides* biting midges in order to better understand the distribution of different species, thereby assisting policy formulation. The Program covers all parts of Portugal including mainland Portugal, the Azores (São Miguel, Santa Maria, Terceira, Graciosa, São Jorge, Pico, Faial, Flores and Corvo islands) and Madeira (Madeira and Porto Santo islands) archipelagos.

*C. imicola* and the Obsoletus and Pulicaris groups are easily identified based on their wing patterns. However, identification of species in these groups to species level, or identification of other species, requires more detailed morphological study or the use of molecular techniques. This paper describes the use of morphological and molecular techniques to identify collections to species level during the ESP from 2005 to 2010 including thirteen *Culicoides* species reported for the first time in mainland Portugal or from Azores archipelago.

## Materials and Methods

### Insect collection

*Culicoides* were collected using miniature CDC light traps (CDC miniature blacklight model 1212, John Hock, USA) fitted with 4W UV bulbs, suction fans and LCS-2 Photoswitch systems. Although Onderstepoort light traps may be more effective at collecting *Culicoides* [10], the miniature CDC light trap is considerably lighter and can be powered by a battery pack rather than requiring a mains electrical connection, making it easier to deploy in remote areas. CDC traps are also effective at attracting mosquitoes, allowing the Surveillance Program to be adapted for mosquito surveillance if required. CDC traps are also used by other national *Culicoides* surveillance schemes in Europe, such as Spain and France, and the use of a consistent trapping protocol facilitates comparison of results across different schemes.

**Figure 1:**
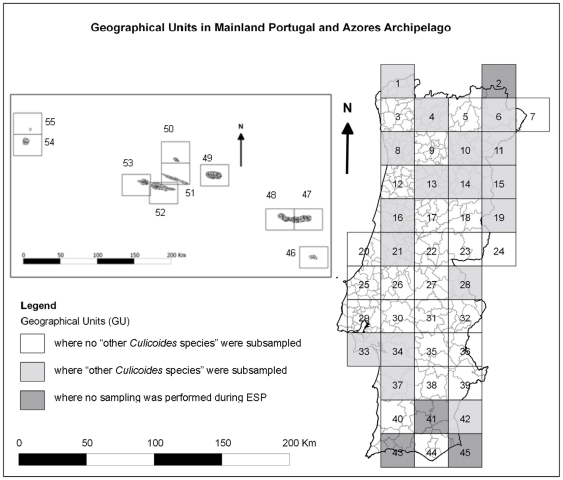
Geographical Units in mainland Portugal and Azores archipelago: 1 GU = 50 km×50 km. Azores archipelago GU – 46: Santa Maria island; 47 and 48: São Miguel island; 49: Terceira island; 50: Graciosa island; 51: São Jorge island; 52: Pico island; 53: Faial island; 54: Flores island; 55: Corvo island.

To ensure systematic coverage, Portugal was divided into 57 geographical units (GUs), each 50 km×50 km (Figure 1). Four of these were not considered to be of epidemiological interest due to low livestock densities and were not sampled. Within each remaining GU, two sheep, goat, cattle or mixed farms with at least five animals were selected. These farms were at least 2.5 km from the coast and separated by at least 10 km. The farms were not allowed to use insecticides during the study.

CDC traps were placed within 30 m of animal enclosures, 1.70 m above ground. The LCS-2 Photoswitch system automatically switched the trap on at dusk and off after dawn. Each trap was operated for one night per week throughout the year. Insects were collected into flasks containing 75% of 70% ethanol and 25% of antifreeze, in a final volume of 500 ml, and were identified at the Interdisciplinary Centre of Research in Animal Health (CIISA) at the Faculty of Veterinary Medicine, Technical University of Lisbon (FMV-UTL).

### Collection of environmental data

Minimum and maximum temperatures and relative humidity were obtained from the closest meteorological station to the trapping site (Annex S1). Maximum temperature was recorded on the day the trap was set and minimum temperature on the day the trap was emptied, to reflect conditions corresponding to the evening/night of collection. Humidity was recorded at 15.00 hours on the day the trap was set and 09.00 hours on the day of collection for the same reason. Wind direction and average speed were recorded on the site on the day of collection.

The presence of livestock species and bodies of water on the farm, human housing and other livestock farms within 10 km was recorded by state veterinary staff during the placement of traps, by completion of a standardized questionnaire. Land cover types in the same parish were extracted from the cartographic farming database maintained by the Institute for Agricultural Financing of the Portuguese Ministry of Agriculture. The proximity of bodies of water, human housing, other farms and vegetation to the trapping site was later verified using aerial photography.

### Morphological identification

Identification of collected *Culicoides* was first attempted using stereoscope microscopy (SM) and wing patterns [11], [12], [13], [14], allowing the identification of *C. imicola* (Kieffer), species from the Obsoletus group [*C. obsoletus* (Meigen), *C. scoticus* (Downes and Kettle), *C. chiopterus* (Meigen) and *C. dewulfi* (Goetghebuer)], *C. pulicaris* (Linnaeus), *C. newsteadi* (Austen), *C. circumscriptus* (Kieffer), *C. punctatus* (Meigen), *C. maritimus* (Kieffer) and *C. univittatus* (Vimmer). This information was supplied to the Central Veterinary Services (CVS) on a weekly basis in order to support policy decisions.

*Culicoides* spp. that could not be identified using keys based on wing pattern and those that were not priority for the ESP for bluetongue (“other *Culicoides* species” in table 1) were preserved in 96% alcohol. Five percent were randomly selected from GUs where at least 65 “other *Culicoides* species” were collected (Figure 1). These specimens were dissected into different body parts (head, thorax, abdomen, wings and legs) using 26 Gauge (0.404 mm diameter) needles, mounted on glass slides using Hoyer's medium and dried in an incubator at 37°C for 3–4 days. Specimens were then examined using composed optical microscopy (COM). Body structures were characterized and measured (eyes, cibarium, palp segments, antennae segments, mouthparts, legs and wings in both sexes, aedeagus, parameres, coxite, style, tergites, lamellae and basal membrane in males, and spermathecae in females). Identification keys based on these morphological characteristics [11], [12], [13], [14] were used to identify specimens to species level.

**Table 1:** Proportion of *Culicoides* species collected on mainland Portugal from 2005 to 2010 and identified solely from wing pattern.

Species	% of total (both sexes)	Presence in Geographical Units^1^
*C. circumscriptus* (Kieffer, 1918)	0.2	3–5; 7; 10; 12; 13; 16–18; 20; 22–40; 42; 44
*C. imicola* (Kieffer, 1913)	75.3	16–18; 20–40; 42; 44
*C. maritimus* (Kieffer, 1924)	0.1	7; 12; 17; 23; 26; 27; 29; 30; 31; 34–36; 38–40; 42; 44
*C. newsteadi* (Austen, 1921)	2.1	1; 3–7; 10; 12–18; 20–40; 42; 44
*C. pulicaris* (Linné, 1758)	0.1	1; 3–10; 12; 16–18; 20–26; 28; 29; 31; 40; 42; 44
*C. punctatus* (Meigen, 1804)	6.4	1; 3–10; 12–18; 20–40; 42; 44
*C. univittatus* (Vimmer, 1932)	<0.1	5; 7; 10; 12; 13; 17; 18; 20; 22–27; 29–31; 34–36; 38–40; 42; 44
Obsoletus group* §	6.5	1; 3–10; 13–18; 20–31; 33–40; 42; 44
Other *Culicoides* species	9.3	1; 3–10; 12; 13; 15–18; 20–40; 42; 44
**Total**	**100**	

1 See Figure 1.

* Includes males and females from *C. obsoletus sensu stricto*, *C. scoticus*, *C. chiopterus* and *C. dewulfi* species (these cannot easily be differentiated by morphology).

§ 280 females were identified to species level via multiplex PCR: *C. obsoletus s.s. -* 69.6%; *C. scoticus* - 30.4%; *C. chiopterus* – 0%; *C. dewulfi* – 0%.

### Molecular identification

In order to measure the relative frequency of species within the Obsoletus group, 288 specimens were selected at random for molecular identification. DNA extraction was performed using the DNeasy Tissue Kit (Qiagen, Crawley, UK) and specimens were identified via multiplex PCR [15]. Amplification was carried out in 50 µl PCR reaction using GeneAmp 1×PCR Gold reaction buffer, 2.5 mM MgCl2 (Applied Biosystems), 200 µM of the four dNTP's (GE Healthcare), 1 µM of each primer: UOAobsF, UOAscoF, UOAchiF, UOAdewF and the common R primer C1-N-2191 [15], 0.5 units of AmpliTaq Gold polymerase (Applied Biosystems) and 5 ng DNA template. The PCR reactions were performed on a Thermal controller (Mastercycler Gradient, Eppendorf) adjusted to the following thermal conditions: DNA denaturation and polymerase activation at 95°C for 10 min, followed by 30 cycles at 95°C for 30 s (denaturation), 61°C for 1 min (annealing), 72°C for 30 s (extension) and 72°C for 5 min (final elongation). The PCR products were subjected to electrophoresis on a 2.0% agarose gel and stained with ethidium bromide for visualization.

## Results

A total of 3.542.652 specimens were collected across mainland Portugal from 2005 to 2010. (Table 1). A summary of the frequency of each of the species or groups identifiable solely on the basis of wing pattern (*C. imicola*, species from the Obsoletus group, *C. pulicaris*, *C. newsteadi*, *C. circumscriptus*, *C. punctatus*, *C. maritimus* and *C. univittatus*) in the total catch, for mainland Portugal, is provided in Table 1.

329.467 specimens (almost 10% of the total) could not be identified solely on the basis of wing pattern or were not a priority for the ESP for bluetongue. Of these specimens, 5% (n = 16.473) were slide-mounted and examined using composed optical microscopy (COM; Table 2). As a result of this, nine *Culicoides* species [*C. alazanicus* (Dzhafarov), *C. bahrainensis* (Boorman), *C. deltus* (Edwards), *C. lupicaris* (Downes and Kettle), *C. picturatus* (Kremer and Deduit), *C. santonicus* (Callot, Kremer, Rault and Bach), *C. semimaculatus* (Clastrier), *C. simulator* (Edwards) and *C. subfagineus* (Delécolle and Ortega)] were reported for the first time in mainland Portugal.

**Table 2:** Frequency and distribution of *Culicoides* species identified in a subsample of 5% of specimens collected in mainland Portugal which could not be identified using the wing pattern keys or were not priority for ESP for bluetongue.

Species	% of total (both sexes)	Presence in Geographical Units^1^
*C. achrayi* (Kettle & Lawson, 1955)	47.3	3; 5; 7; 9; 17; 18; 25; 39
*C. alazanicus* X (Dzhafarov, 1961)	1.2	40
*C. bahrainensis* X (Boorman, 1989)	0.2	36; 38
*C. corsicus* (Kremer, Leberre & Beaucournu-S., 1971)	0.1	23; 35; 36
*C. deltus* X (Edwards, 1979)	0.2	9
*C. fascipennis* (Staeger, 1839)	3.8	5; 7; 9; 12; 18; 25; 26; 30; 39
*C. festivipennis* (Kieffer, 1914)	5.8	7; 12; 18; 23; 25; 30; 35; 40
*C. gejgelensis* (Dzhafarov, 1964)	6.7	7; 12; 18; 20; 22; 23; 26; 30; 36; 44
*C. heliophilus* (Edwards, 1921)	0.2	18
*C. heteroclitus* (Kremer & Callot, 1965)	3.3	7; 9; 25
*C. impunctatus* (Goetghebuer, 1920)	0.1	4; 20
*C. indistinctus* (Khalaf, 1961)	0.6	7; 9; 18; 25; 32; 35
*C. jumineri* (Callot & Kremer, 1969)	2.6	5; 7; 17; 18; 23; 32; 35; 38; 39
*C. jurensis* (Callot, Kremer & Déduit, 1962)	0.2	30; 36; 39
*C. kibunensis* (Tokunaga, 1937)	0.4	23
*C. longipennis* (Khalaf, 1957)	5.1	7; 23; 32; 35; 39
*C. lupicaris* X (Downes & Kettle, 1952)	0.1	25
*C. nubeculosus* (Meigen, 1930)	0.4	23; 27; 29; 40
*C. odiatus* (Austen, 1921)	0.1	9; 18; 23; 30
*C. parroti* (Kieffer, 1922)	1.9	23; 39
*C. picturatus* X (Kremer & Déduit, 1961)	1.7	23
*C. pseudopallidus* (Khalaf, 1961)	1.0	7; 23; 35; 38
*C. puncticollis* (Becker, 1903)	0.9	23; 32
*C. sahariensis* (Kieffer, 1923)	0.1	39
*C. santonicus* X (Callot, Kremer, Rault & Bach, 1968)	11.5	7; 18; 22–24; 40
*C. semimaculatus* X (Clastrier, 1958)	0.1	40
*C. simulator* X (Edwards, 1939)	0.2	7
*C. subfagineus* X (Delécolle & Ortega, 1998)	0.7	7; 18; 30; 31; 40
*C. subfascipennis* (Kieffer, 1919)	2.1	7; 23; 25; 30; 32; 39
*C. vexans* (Staeger, 1839)	1.8	7
**Total**	**100**	

X Species reported for the first time in mainland Portugal.

A total of 280.288 female specimens of the Obsoletus group were identified to species level via multiplex PCR. 69.6% (n = 200) of these were *C. obsoletus sensu strictu*, 30.4% (n = 88) were *C. scoticus*, but no *C. chiopterus* or *C. dewulfi* were detected.

A total of 30.879 specimens were collected in the Azores from 2005 to 2010 (Tables 3 and 4). Two species not previously reported were identified (*C. circumscriptus* and *C. newsteadi*). Eight females within the Obsoletus group were identified by multiplex PCR as *Culicoides scoticus*, a species not previously reported from the archipelago.

**Table 3:** Total *Culicoides* species reported in the islands of Azores Archipelago (N = 30879 specimens).

Species	% of total (both sexes)	Geographical Units^1^
*C. circumscriptus*	2.5	46–53
*C. newsteadi*	0.8	46–52
Obsoletus group* §	96.7	46–55
**Total**	**100**	

* Includes males and females from *C. obsoletus s. s.*, *C. scoticus*, *C. chiopterus* and *C. dewulfi* species (these cannot easily be differentiated by morphology).

§ 8 females were identified to species level by multiplex PCR: *C. obsoletus s.s. -* 0%; *C. scoticus* - 100%; *C. chiopterus -* 0%; *C. dewulfi* – 0%.

**Table 4:** Culicoides species reported in the nine Islands of Azores Archipelago (N = 30879 specimens).

Species	Islands of Azores Archipelago																	
	Santa Maria		São Miguel		Terceira		Graciosa		São Jorge		Pico		Faial		Flores		Corvo	
	A	B	A	B	A	B	A	B	A	B	A	B	A	B	A	B	A	B
Obsoletus Group†	***29-10-08***	81.29	***<09-05***	89.07	***<09-05***	77.37	***16-11-08***	71.43	***<09-05***	99.65	***<09-05***	97.42	***<09-05***	70.59	***13-08-08***	99.8	***29-09-09***	83.3
*C. scoticus* §	***06-09-10***	0.05*	***31-08-08***	0.21	***22-06-09***	2.19	***18-12-09***	7.14*	***13-10-10***	0.01*	***03-11-08***	0.04**	***19-11-08***	0.9	***11-08-10***	0.2*	***16-12-09***	16.7*
*C. newsteadi*	***28-10-08***	11.48	***19-10-05***	1.44	***01-07-08***	5.84	***03-12-09***	7.14	***20-07-10***	0.01	***06-07-11***	0.01	NR		NR		NR	
*C. circumscriptus*	***29-10-08***	7.19	***19-10-05***	9.28	***28-07-10***	14.6	***02-12-09***	14.29	***27-07-10***	0.33	***20-09-09***	2.54	***04-08-09***	28.51	NR		NR	

A – Date of the first specimen identification; B – Percentage (of the total) of *Culicoides* specimens found in the island until November 2010; NR – Not reported;

† Obsoletus Group include the females of the *C. obsoletus s.s.*, *C. scoticus*, *C. chiopterus* and *C. dewulfi* species;

§ All the specimens found are referred to the male genre, except

* where 5 females were identified based on morphological features and

** where 8 females where identified by multiplex PCR [15]. The dates in normal writing refer to species that were first identified by other authors until September 2005 [23]. The dates in bold and italic refer to the first mention of the species in the island by the ESP.

Table 5 shows the results of the environmental surveys for the sites from which each of the species previously unreported from mainland Portugal was detected, while table 6 shows the same for the species previously unreported from Azores. Information on the ecology of these species from other sources is given in Annex S2 and Annex S3 [16], [17], [18], [19], [20]. Table 7 shows the meteorological data collected from the sites in mainland Portugal at which the previously unreported species were found.

**Table 5:** Ecological data and characterization of the sampling place and surroundings (mainland Portugal).

Species	Sampling Date	Environment near the sampling place (Original data collected in mainland Portugal based on surveys)				Animals in sampling place
		Vegetation (Distance in meters)	Water (Distance in meters)	Human housing (Distance in meters)	Farms (Distance in meters)	
*C. alazanicus*	**2008**: August **2010**: April	Pastures, Upland, Undergrowth, Irrigation, Cork-trees, Holmoaks, Olive-trees(140)	Absent on surroundings (>10000)	Present (3300)	Absent on surroundings (>10000)	Cattle, Sheep, Goat
*C. bahrainensis*	**2005**: June and August	Pastures, Upland, Orange-trees, Figs and Undergrowth (0)	Drip irrigation, Lagoon (480–1024)	Present (1470–2047)	Present (1000)	Cattle, Sheep, Goat
*C. deltus*	**2010**: May and June	Present (500)	Lagoon (430)	Present (360)	Present (360)	Cattle
*C. lupicaris*	**2010**: March	Pine-trees, Undergrowth (0)	Lagoon (400)	Present (2000)	Present (380)	Cattle
*C. picturatus*	**2010**: April, May	Trees and other vegetation (900)	Lagoon, River and Dam (241–9080)	Present (4390)	Present (9000)	Cattle
*C. santonicus*	**2010**: March, April, May	Pastures, Upland, Irrigation, Cork-trees, Olive-trees, Oak-trees, Holmoaks and Undergrowth(0–300)	River, Lake, Streams, Reeds, Dams, Lagoon (100–5100)	Present (620–5100)	Present (500–610)	Cattle, Sheep, Goat
*C. semimaculatus*	**2008**: July	Streams, Reeds, Dams, Pastures, Upland, Undergrowth, Irrigation, Cork-trees, Holmoaks, Olive-trees(140)	Absent on surroundings (>10000)	Present (3300)	Absent on surroundings (>10000)	Cattle, Sheep, Goat
*C. simulator*	**2010**: May	Apple-trees, Oak-trees, Chestnuts (0)	Lagoon (400)	Present (1000)	Present (510)	Cattle
*C. subfagineus*	**2008**: July and August; **2009**: May; **2010**: April, May, August and November	Pastures, Upland, Irrigation, Cork-trees, Holmoaks, Olive-trees, Pine-trees, Cherry-trees, and Undergrowth (200–400)	Streams, Reeds, Dams, Lagoon, Padies (190–350)	Present (0–1610)	Present (150–500)	Cattle, Sheep, Goat

**Table 6:** Ecological data and characterization of the sampling place and surroundings (Azores archipelago).

Species	Sampling Date	Environment near the sampling place (Original data collected in Azores archipelago based on questionnaire)				Animals in sampling place
		Vegetation	Water	Human housing	Farms	
*C. circumscriptus*	**2005**: October and November; **2008**: March and October; **2009**: August, October to December; **2010**: June to September	Pastures; Upland; Irrigation; Laurel, forests, mountains, cryptomerias, incense, plane trees, acacia, sweet pittosporum, eucalyptus, beech, heather, orchard	Present (Lagoon; Reeds)	Present	Present	Cattle
*C. newsteadi*	**2005**: October and November; **2008**: July, October and November; **2009**: June, November and December; **2010**: June to August	Pastures; Upland; Irrigation; Laurel, forests, mountains, cryptomerias, incense, plane trees, acacia, sweet pittosporum, eucalyptus, beech, heather	Present (Lagoon)	Present	Present	Cattle
*C. obsoletus sensu stricto*	**2005**: October and November; **2008**: March, July to November; **2009**: June, August to December; **2010**: June to October	Pasture; Upland; Irrigation; Laurel, forests, mountains, cryptomerias, incense, plane trees, acacia, sweet pittosporum, eucalyptus, beech, heather, orchard, poplar, willow fennel	Present (Lagoon; Reeds)	Present	Present	Cattle
*C. scoticus*	**2008**: March and November; **2009**: June	Pastures; Upland; cryptomerias, incense, plane trees, acacia, sweet pittosporum, beech, incense, laurel, forests, orchard	Present (Lagoon; Reeds)	Present	Present	Cattle

**Table 7:** Meteorological data for the different sampling captures in mainland Portugal.

Species	Sampling Day	Tmin (°C)*	Tmax (°C)*	Wind Direction	Average Wind Speed (Km/h)	Relative Humidity 15 h–9 h (%)
*C. alazanicus*	06-04-2010	9.6/11	21.8/19	Northwest	12.6	28–68
	12-08-2008	18.6/14	24.9/28	Northwest	21.6	74–100
*C. bahrainensis*	02-08-2005	16.3/19	31.6/29	North	13.3	23–37
	08-06-2005	19.1/14	36.4/37	South	12.5	29–38
*C. deltus*	01-06-2010	15.6/18	24.8/23	Variable	9	72–71
	19-05-2010	14.4/17	26.5/20	East	12.2	40–38
*C. lupicaris*	15-03-2010	5.2/−	15.3/−	Northeast	6.8	37–62
*C. picturatus*	26-04-2010	10.8/13	25.7/21	Unknown	Unknown	40–71
	03-05-2010	8.2/8	22/20	North	17.6	44–52
*C. santonicus*	26-04-2010	9.9/13	18.9/21	Southeast	11.9	60–64
	13-04-2010	6.9/12	18.2/20	Northeast	11.9	42–61
	31-03-2010	2.3/8	13.3/18	Southwest	9	59–80
	21-04-2010	14.7/16	23.2/23	South	11.2	64–67
	26-04-2010	9.9/13	18.9/21	Southeast	11.9	60–64
	24-05-2010	13.2/6	30.1/18	Southeast	13.7	32–61
*C. semimaculatus*	29-07-2008	14.7/17	23.6/29	Northwest	23	72–73
*C. simulator*	24-05-2010	13.2/6	30.1/18	Southeast	13.7	32–61
*C. subfagineus*	18-08-2010	14.3/19	31.2/27	West	8.6	24–49
	19-04-2010	10.8/11	15.8/22	Northeast	1.4	84–92
	04-11-2010	10.7/8	23/24	Northeast	9	47–73
	26-05-2009	10.3/8	16.3/24	North	17.3	71–62
	06-05-2010	9.4/7	19.3/23	West	11.2	37–60
	29-07-2008	14.7/17	23.6/29	Northwest	23	72–73
	12-08-2008	18.6/14	24.9/28	Northwest	21.6	74–100

* **Temperatures from meteorological stations/local of sampling.**

**Figure 2:**
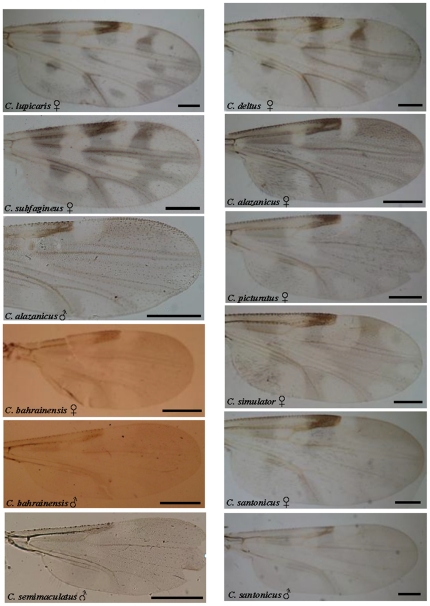
Photographs of the wings of the nine *Culicoides* species reported for the first time in mainland Portugal (*C. lupicaris*, *C. deltus*, *C. subfagineus*, *C.alazanicus*, *C. picturatus*, *C. bahrainensis*, *C. simulator*, *C. santonicus*, *C. semimaculatus*) in this study. Bar = 200 µm.

**Figure 3 pone-0034896-g003:**
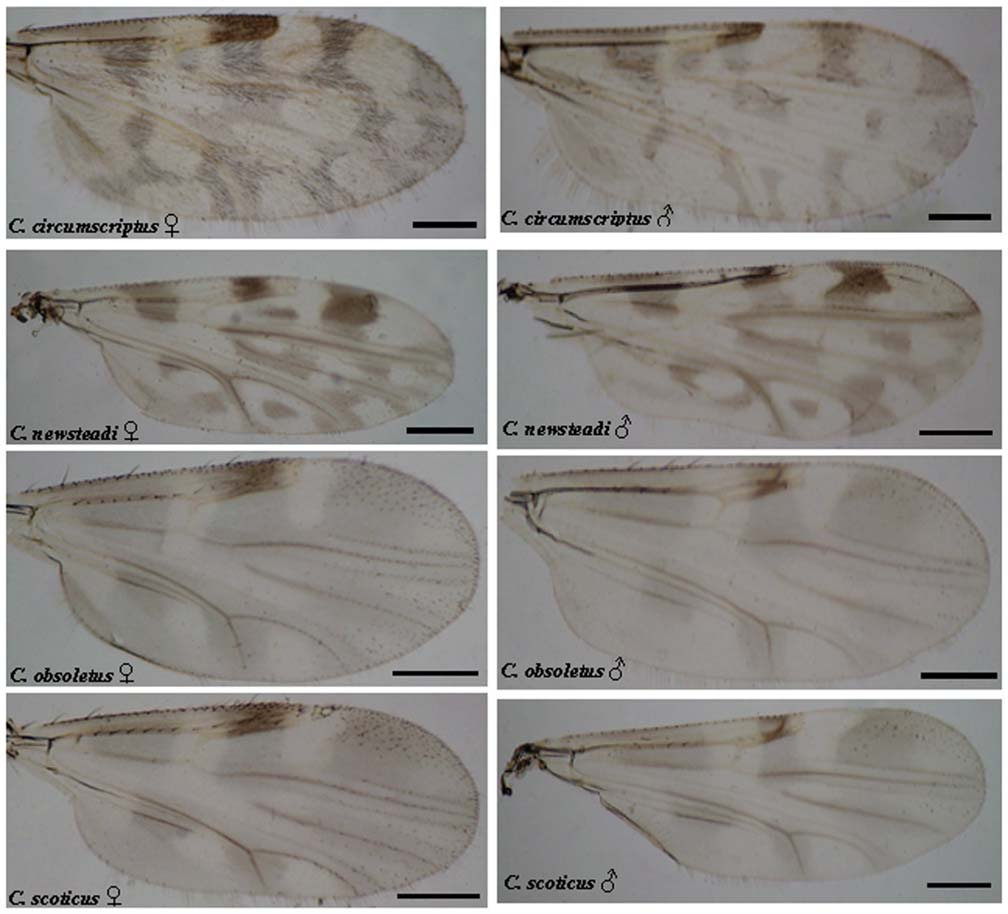
Photographs of the wings of the four *Culicoides* species reported for the first time in Azores archipelago (*C. circumscriptus, C. newsteadi, C. obsoletus, C. scoticus*) in this study. Bar  =  200µm.

Measurements (mean values) of different body structures recorded for each of the species previously unreported from mainland Portugal and Azores are given in Table 8 and Table 9 respectively. Table 10 shows their distribution in other European and Mediterranean countries. Finally, Figure 2 and 3 show the wing pattern of the *Culicoides* species reported for the first time in this study.[Fig pone-0034896-g003]

**Table 8:** Measurement (mean values) of the nine *Culicoides* species first reported in mainland Portugal.

Species	Date of the first randomly sample selected	Wing			Palp			Antennae			Spermathecae	
		Length (µm)	Width (µm)	Costa (µm)	Length (µm)	Ratio 3/(1+2)	Third palp segment length (µm)	Length (µm)	Antennary Index	Ratio	Number	Length (µm)
*C. lupicaris* (♀)	18-03-2010	1975	862.5	1250	350	0.73	110	1062.5	1.34^*^	1.45^***^	2	First: 90; Second: 68.13
*C. subfagineus* (♀)	01-08-2008	1189.1	523.9	734.4	235.3	1.11	90	701.1	0.9^*^	1.26^***^	2	First: 62.03; Second: 58.58
*C. picturatus* (♀)	05-05-2010	1286.7	596	747.8	220	0.93	76.1	626.2	1.04^*^	1.4^***^	2	First: 74.29;Second: 69.55
*C. alazanicus* (♀)	22-08-2008	1085.4	473.6	625.7	170.8	1.38	72.2	622.4	1.52^*^	2.04^***^	2	First: 47.85;Second: 43.54
*C. alazanicus* (♂)	28-08-2008	1000	360	550	136.25	1.33	50	725	0.77^**^	2.89^****^	n.a.	n.a.
*C. santonicus* (♀)	08-04-2010	1745.2	789.4	1020.7	311.25	0.92	107.7	781.4	1.19^*^	1.5^***^	2	First: 69.9;Second: 65.34
*C. santonicus* (♂)	22-04-2010	1684.4	609.4	897	232.5	1.11	80	971.9	0.735^**^	3.07^****^	n.a.	n.a.
*C. deltus* (♀)	28-05-2010	1712.5	787.5	1093.8	320	1.24	126.25	790.6	0.94^*^	1.25^***^	2	First: 75.63;Second: 64.38
*C. simulator* (♀)	27-07-2010	1537.5	675	862.5	248.8	1.13	101.3	750	1.23^*^	1.62^***^	2	First: 66.25;Second: 54.38
*C. bahrainensis* (♀)	02-08-2005	900	450	520	175	1.21	72.5	533.75	1.21^*^	1.4^***^	2	First: 60;Second: 50
*C. bahrainensis* (♂)	08-06-2005	1020	390	520	143.8	0.95	51.25	687.5	0.72^**^	3.1^****^	n.a.	n.a.
*C. semimaculatus* (♂)	01-08-2008	740	290	355	158.75	0.97	40	520	0.71^**^	2.83^****^	n.a.	n.a.

♀ = Female; ♂ = Male; Costa = Length of the wing from *arculus* to the terminus of second radial cell; Ratio 3/(1+2) = Length of the third palp segment/Length of the first and second palp segments; Antennary Index = *Length of eleventh to fifteenth antennae segments/Length of the third to tenth antennae segments and **Length of thirteenth to fifteenth antennae segments/Length of the third to twelfth antennae segments; Ratio = ***Length of the eleventh antennae segment/Length of the tenth antennae segment and ****Length of the thirteenth antennae segment/Length of the twelfth antennae segment. First spermatheca: mean value of the biggest spermatheca of different specimens; Second spermatheca: mean value of the smallest spermatheca of different specimens; n.a. = Not applicable.

**Table 9:** Measurement (mean values) of the four *Culicoides* species first reported in Azores archipelago.

Species	Date of the first randomly sample selected	Wing			Palp			Antennae			Spermathecae	
		Length (µm)	Width (µm)	Costa (µm)	Length (µm)	Ratio 3/(1+2)	Third palp segment length (µm)	Length (µm)	Antennary Index	Ratio	Number	Length (µm)
*C. circumscriptus* (♀)	19-10-2005	1540.8	689.2	898.3	255.4	1.07	96.6	747.3	1.38^*^	1.92^***^	1	106.3
*C. circumscriptus* (♂)	07-11-2005	1379.5	505.4	733.9	201.5	1.05	67.5	879.5	0.7^**^	2.49^****^	n.a.	n.a.
*C. newsteadi* (♀)	19-10-2005	1230	520	716.3	200.5	1.09	75.2	608.5	0.94^*^	1.29^***^	2	First: 66.52Second: 60.88
*C. newsteadi* (♂)	25-11-2008	1087.5	383.3	612.5	161.7	1.14	55	705	0.61^**^	2.49^****^	n.a.	n.a.
*C. obsoletus sensu stricto* (♀)	19-10-2005	1062.5	510.2	648.4	165.6	0.88	51.1	546.9	1.03^*^	1.24^***^	2	First: 49.14Second: 43.91
*C. obsoletus sensu stricto* (♂)	07-11-2005	1162.5	406.3	693.8	162.8	0.89	48.8	757.5	0.71^**^	2.97^****^	n.a.	n.a.
*C. scoticus* (♀)	16-12-2009	1040.6	481.3	603.1	161.7	0.86	50.8	505.8	1^*^	1.45^***^	2	First: 68.33Second: 63.13
*C. scoticus* (♂)	31-03-2008	1227.5	443.8	696.3	153.8	0.81	41.75	838.8	0.71^**^	2.96^****^	n.a.	n.a.

♀ = Female; ♂ = Male; Costa = Length of the wing from *arculus* to the terminus of second radial cell; Ratio 3/(1+2) = Length of the third palp segment/Length of the first and second palp segments; Antennary Index = *Length of eleventh to fifteenth antennae segments/Length of the third to tenth antennae segments and **Length of thirteenth to fifteenth antennae segments/Length of the third to twelfth antennae segments; Ratio = ***Length of the eleventh antennae segment/Length of the tenth antennae segment and ****Length of the thirteenth antennae segment/Length of the twelfth antennae segment. First spermatheca: mean value of the biggest spermatheca of different specimens; Second spermatheca: mean value of the smallest spermatheca of different specimens; n.a. = Not applicable.

**Table 10:** *Culicoides* species first reported in mainland Portugal and Azores archipelago and their presence in other European and Mediterranean countries.

Species	Female	Male	Region of Portugal	Number of specimens found	Other Countries^3^		
					SEC/MC	CEC	NEC
*C. (Culicoides) lupicaris* (Downes & Kettle, 1952)	**X**		**Mainland Portugal**	**1** 1	**X**	**X**	**X**
*C. (Culicoides) subfagineus* (Delécolle & Ortega, 1998)	**X**		**Mainland Portugal**	**10** 1	**X**		
*C. (Silvaticulicoides) picturatus* (Kremer & Déduit, 1961)	**X**		**Mainland Portugal**	**23** 1	**X**	**X**	**X**
*C. (Synhelea) semimaculatus* (Clastrier, 1958)		**X**	**Mainland Portugal**	**1** 1	**X**	**X**	
*C. alazanicus* (Dzhafarov, 1961) (syn. *C. musilator*)	**X**	**X**	**Mainland Portugal**	**16** 1	**X**	**X**	**X**
*C. santonicus* (Callot, Kremer, Rault & Bach, 1968)	**X**	**X**	**Mainland Portugal**	**154** 1	**X**		
*C. (Culicoides) deltus* (Edwards, 1939)	**X**		**Mainland Portugal**	**3** 1	**X**	**X**	**X**
*C. simulator* (Edwards, 1939)	**X**		**Mainland Portugal**	**2** 1	**X**	**X**	**X**
*C. baharainensis* (Boorman, 1989)	**X**	**X**	**Mainland Portugal**	**2** 1	**X**		
*C. (Beltranmyia) circumscriptus* (Kieffer, 1918)	**X**	**X**	**Azores archipelago**	**784** 2	**X**	**X**	**X**
*C. (Culicoides) newsteadi* (Austen, 1921)	**X**	**X**	**Azores archipelago**	**247** 2	**X**	**X**	**X**
*C. (Avaritia) obsoletus* (Meigen, 1818)	**X**	**X**	**Azores archipelago**	**11** * 2	**X**	**X**	**X**
*C. (Avaritia) scoticus* (Downes & Kettle, 1952)	**X**	**X**	**Azores archipelago**	**19** * 2	**X**	**X**	**X**

Syn. – Synonymy;

1 Since the first specimen identified until the beginning of November 2010;

2 Since the first specimen until July 2011;

3 According to [14], [24], [25];

* From a total of 29844 midges belonging to the Obsoletus Group, where females of *C. obsoletus s.s.*, *C. scoticus*, *C. chiopterus* and *C. dewulfi* are included. SEC/MC – South European and Mediterranean Countries (Portuguese mainland; Spain; Andorra; France; Corsica; Italy; Sicily; Greek mainland; Croatia; Bulgaria; Morocco; Algeria; Tunisia; Cyprus; North Aegean Islands; Dodecanese Islands; Turkey); CEC – Central European Countries (Netherlands; Belgium; Germany; Switzerland; Austria; Romania; Czech Republic; Slovakia; Bosnia and Herzegovina; Ukraine; Poland; Romania; Hungary; Central Russia); NEC – North European Countries [Belarus; Estonia; United Kingdom; Ireland (incl. North Ireland); Sweden; Denmark; Norwegian mainland; Lithuania; Latvia; Northwest Russia].

## Discussion

The objective of this study was to improve knowledge of the *Culicoides* fauna present in Portugal, both on the mainland and islands. Other *Culicoides* surveys have previously been conducted in mainland Portugal [12], [21], [22], but using a smaller number of sites, for a shorter period and not including Azores archipelago. The national ESP described here therefore represents the largest survey of *Culicoides* ever conducted in Portugal.

As a result of our study, nine *Culicoides* species were detected for the first time in mainland Portugal. As can be seen from Table 1, the species most commonly associated with farms in mainland Portugal was *C. imicola* (75.3%), which is an important vector of BTV and African horse sickness virus. *Culicoides* species within Obsoletus group, also important for BT and AHS epidemiology, were also common (6.5% of total catch).

Our study also detected three new species in the Azores archipelago. *C. newsteadi* and *C. circumscriptus* were identified for the first time and were found on multiple islands (*C. newsteadi* in São Miguel, Santa Maria, Terceira, Graciosa and São Jorge and *C. circumscriptus* in Santa Maria, São Miguel, Terceira, Graciosa, São Jorge, Pico and Faial). *C. scoticus*, a member of Obsoletus group, was also reported from the archipelago for the first time and was detected on all nine islands. Although Obsoletus-group *Culicoides* have previously been reported from several islands, including São Miguel, Terceira, São Jorge, Pico and Faial [23], no attempt had previously been made to identify collections to species level. The role played by Obsoletus-group *Culicoides* in the recent outbreak of BTV-8 in northern Europe increases the value of information about the distribution of species in the Obsoletus group. Our results not only extend the known distribution of species within the Obsoletus group to all islands of Azores archipelago, but also provide the first species-specific information about *Culicoides* within the Obsoletus group for the Azores. The recent development of molecular techniques for species-level identification of females belonging to Obsoletus group [15] is likely to result in data about the epidemiological role of individual species in this cryptic group in the near future.

Although it was not the primary aim of this study, new data were acquired on the seasonal activity, habitats and environmental requirements of Portuguese *Culicoides* species. *C. alazanicus* was found in April and August, while previous studies elsewhere in Europe have suggested its seasonal activity to be restricted to the months of June and July [16]. Furthermore, although the same study suggested that this species requires proximity to bodies of water for larval development, we confirmed the presence of the species at a site where no substantial water sources were present within at least 10 km.

*C. bahrainensis* has previously been reported from the Mediterranean basin and North Africa. On one occasion we collected the species under conditions of relatively low humidity (23–37%). Previous studies in Saudi Arabia have suggested that the absence of this species between July and August and its high density in April and November may indicate a relatively low tolerance for dry conditions [17]. Our findings indicate that other factors may also be involved. Alternatively, the presence of water sources near our sampling site and the high wind speed (12.5 to 13.3 km/h) during the collection period may have resulted in the introduction of the species into our catch.

*C. deltus* has been previously reported from many sites across Europe. We detected the species during May and June and in proximity to cattle, in common with results from other published studies [18].

*C. lupicaris* was found in only one sample in March 2010. The habitat close to the collection site was similar to that reported in previous studies which have detected this species [19], including the presence of a lagoon, representing a suitable habitat for larval development and adult survival. The species was active under relatively cool conditions; the temperature recorded during the night of collection varied from 5.2°C to 15.3°C.

*C. picturatus* was also found at sites in proximity to bodies of water, as might be expected from its preference for marshy non-saline habitats [18]. We detected this species from collections made in April and May, with temperatures ranging between 8°C and 25.7°C. Little has previously been published on the seasonal activity of this species.

*C. santonicus* was the most common of the species detected for the first time in mainland Portugal during this study, with 154 specimens identified until the beginning of November 2010 (Table 10). This species has been recorded from a number of other sites in southern Europe [14], [24], [25]. Sites of large collections corresponded to regions of high farm density, which may help to explain the high number of specimens found. The species was found between March and May, with relative humidity varying between 32% and 80% and with temperatures ranging from 2.3°C to 30.1°C. The local environment at the sites where this species was recorded included a high abundance of water sources and a variety of vegetation types (Table 5), together with the presence of cattle, goats and sheep.

*C. semimaculatus* has previously been reported from southern Europe but not from northern Europe (Table 10). We detected the species at temperatures ranging from 14.7°C to 29°C and at a relative humidity of 72% to 73%, in proximity to cattle, goats and sheep.

*C. simulator* was found only in May and in proximity to various types of trees, similar to previously reported habitats [18]. A lagoon near the sampling place may represent a larval breeding site for this species. Cattle were present on the farm where trapping was conducted.

*C. subfagineus* was found between April and November, making it the species with the widest period of seasonal activity of those newly discovered in mainland Portugal. The species has previously been reported from various sites in southern Europe [14], [24], [25] and North Africa. This result therefore increases the known geographical range of the species but does not represent an unusual environment in which to find it.

*C. alazanicus*, *C. semimaculatus* and *C. bahrainensis* were only found in southern regions of mainland Portugal. These species have previously been reported from a number of countries in southern Europe and the Mediterranean. *C. subfagineus*, *C. santonicus* and *C. bahrainensis* were not previously found in central and northern European countries. Of the species newly detected during our study, only *C. alazanicus* and *C. semimaculatus* were found at sites further than 10 km from water bodies (Table 5). Many species of *Culicoides* however thrive in relatively dry conditions and in microclimates often found on farms such as drinking troughs and fresh manure, with a level of moisture sufficient for their development.

Our study also increased our knowledge about the presence and distribution of *Culicoides* species in the Azores. From the four species identified, three were previously unknown from Azores archipelago.

*C. circumscriptus* and *C. scoticus*, as well as a large number of specimens from Obsoletus group unidentified to species level, were recorded from March through to December, while *C. newsteadi* was found only from June to December. The climate of the Azores is warm and humid, making it suitable for the development and survival of many species of *Culicoides*, and those species which have historically been introduced to the islands are in some cases present in high numbers (Table 10). Nevertheless, the diversity of *Culicoides* species present was very low compared to mainland Portugal. This is probably because of the distance of the islands from the continent and the resulting low likelihood of *Culicoides* being introduced via the wind, a hypothesis also supported by the fact that the number of *Culicoides* species present on the islands declines from east to west (with only two species present on the two islands on the west of the archipelago). Our findings are therefore entirely what would be predicted by the theory of island biogeography.

The morphological measurements made during this study (Table 8, Table 9) are similar to those performed by Delécolle and Pena (Annex S4, Annex S5, Annex S6), although some variation exists. This may be due to intraspecific variation or could alternatively be the result of different environmental conditions in breeding sites. Our study provides new information on the *Culicoides* species present in mainland Portugal and in the Azores, as well as on their seasonal activity and spatial distribution. The outputs of this study will be used to inform policy decisions. Confirmation of the presence of a potential vector of BT disease across the Azores archipelago also emphasizes the importance of veterinary education and surveillance measures to minimize the impact of future incursions of BTV. The data may also be of value for future studies, to identify habitat or host preferences of the species described. Due to constraints of time and funding, only a small percentage of samples could be subjected to morphological or molecular analysis; further study could reveal additional information about seasonal activity, interannual variation and habitat preferences.

## Supporting Information

Annex S1 **Distance between farms where nine** ***Culicoides*** **species were reported for the first time in mainland Portugal and the closest meteorological station.**
(DOC)

Annex S2 **Ecological data and characterization of the sampling place and surroundings for the species first reported in mainland Portugal.**
(DOC)

Annex S3 **Ecological data and characterization of the sampling place and surroundings for the species first reported in Azores archipelago.**
(DOC)

Annex S4 **Measurements (mean values) performed by Delécolle (1985) of four** ***Culicoides*** **species reported for the first time in mainland Portugal.**
(DOC)

Annex S5 **Measurements (mean values) performed by Delécolle (1985) of four** ***Culicoides*** **species reported for the first time in Azores archipelago.**
(DOC)

Annex S6 **Measurements (mean values) performed by Pena (2003) of four** ***Culicoides*** **species reported for the first time in Azores archipelago.**
(DOC)
