# Supplementary material for: First Report of 13 Species of Culicoides (Diptera: Ceratopogonidae) in Mainland Portugal and Azores by Morphological and Molecular Characterization
Source: PLoS One. 2012 Apr 19;7(4):e34896. doi: 10.1371/journal.pone.0034896 (PMC3334969; doi:10.1371/journal.pone.0034896)
Supplement: Annex S1 — Distance between farms where nine Culicoides species were reported for the first time in mainland Portugal and the closest meteorological station. (DOC) [file pone.0034896.s001.doc]

| **Species** | **Geographical Unit^1^** | **Geographical Coordinates of Farms** | **Distance in straight line (km)** |
| --- | --- | --- | --- |
| *C. alazanicus*; *C. santonicus*; *C. semimaculatus*; *C. subfagineus* | 40 | **37° 34' 31"N, 8° 36’ 3"W** | 47,47 |
| *C. bahrainensis* | 36 | **38° 24' 27"N, 7° 33' 13"W** | 35,92 |
| *C. bahrainensis* | 38 | **38° 2' 6"N, 7° 53' 54"W** | 3,50 |
| *C. deltus* | 9 | **41° 15' 55"N, 8° 00' 05"W** | 52,61 |
| *C. lupicaris* | 25 | **39° 52' 9"N, 8° 46' 7"W** | 59,38 |
| *C. picturatus; C. santonicus* | 23 | **40° 53' 40"N, 7° 16' 24"W** | 73,60 |
| *C. santonicus* | 18 | **40° 5' 49"N, 7° 25' 20"W** | 19,01 |
| *C. santonicus* | 24 | **39° 53' 32"N, 6° 58' 21"W** | 63,80 |
| *C. santonicus* | 22 | **39° 40' 11"N, 7° 38' 50"W** | 68,13 |
| *C. santonicus*; *C. simulator* | 7 | **41° 23' 59"N, 6° 26' 22"W** | 50,48 |
| *C. subfagineus* | 7 | **41° 11' 13"N, 6° 43' 27"W** | 68,98 |
| *C. subfagineus* | 18 | **40° 23' 16"N, 7° 15' 7"W** | 21,65 |
| *C. subfagineus* | 30 | **38° 59' 40"N, 8° 27' 48"W** | 69,16 |
| *C. subfagineus* | 31 | **39° 2' 36"N, 7° 53' 36"W** | 47,57 |

^1^See Figure 1 for detailed information
