# Supplementary material for: First Report of 13 Species of Culicoides (Diptera: Ceratopogonidae) in Mainland Portugal and Azores by Morphological and Molecular Characterization
Source: PLoS One. 2012 Apr 19;7(4):e34896. doi: 10.1371/journal.pone.0034896 (PMC3334969; doi:10.1371/journal.pone.0034896)
Supplement: Annex S2 — Ecological data and characterization of the sampling place and surroundings for the species first reported in mainland Portugal. (DOC) [file pone.0034896.s002.doc]

| **Species** | **Habitat** | **Host Range** |
| --- | --- | --- |
|
| *C. alazanicus* | The larva was described by Glukhova (1979), and it was bred from mud along a canal by Kremer & Callot (1961)***  Marsh-forest*; June to July* | I.N.A. |
| *C. bahrainensis* | Requires high humidity for survival and development**  Higher peak in April; lower peak in November (T=22,4°C - 29,3ºC, HR = 34-66%); No captures between July-August (T= 33ºC - 38ºC; HR = 24%-54%)** | I.N.A. |
| *C. deltus* | March to August* | The adults have been recorded biting horses and cattle***; goats***** |
| *C. lupicaris* | Oak-trees and Pastures****; April to September* | The adults have been recorded biting horses and cattle***; goats***** |
| *C. picturatus* | Bred from marshy non-saline habitats; little else known*** | I.N.A. |
| *C. santonicus* | I.N.A. | I.N.A. |
| *C. semimaculatus* | I.N.A. | I.N.A. |
| *C. simulator* | Nothing known, but probably a woodland species*** | I.N.A. |
| *C. subfagineus* | I.N.A. | Nothing known; the type series are from light traps*** |

*According to [16]; **According to [17]; ***According to [18]; ****According to [19]; *****According to [20]; I.N.A. = Information not available
