# Supplementary material for: First Report of 13 Species of Culicoides (Diptera: Ceratopogonidae) in Mainland Portugal and Azores by Morphological and Molecular Characterization
Source: PLoS One. 2012 Apr 19;7(4):e34896. doi: 10.1371/journal.pone.0034896 (PMC3334969; doi:10.1371/journal.pone.0034896)
Supplement: Annex S3 — Ecological data and characterization of the sampling place and surroundings for the species first reported in Azores archipelago. (DOC) [file pone.0034896.s003.doc]

| **Species** | **Habitat** | **Host Range** |
| --- | --- | --- |
|
| *C. circumscriptus* | Larvae have been found in mud rich in organic matter and also in puddles in dune sand in Israel; Probably is autogenous for at least its first egg batch* | Probably birds; Females were collected in horses, buffaloes and toads* |
| *C. newsteadi* | Found from May to October; Swarming behaviour* | Fierce man-biter species* |
| *C. obsoletus sensu stricto* | Bred from horse dung and compost heaps, but not from cow dung; Females may be taken during winter months occasionally, but it mainly overwinters as a larvae; It’s one of the first species to appear in the Spring; Tree holes and moist forest leaf litter* | Man-biting pest; Important pest of livestock* |
| *C. scoticus* | This species often occurs with *C. obsoletus s.s.* It has been bred from fungi; Probably breeds in the same habitats as *C. obsoletus sensu stricto** | Bites cattle* |

*According to [18].
