# Supplementary material for: First Report of 13 Species of Culicoides (Diptera: Ceratopogonidae) in Mainland Portugal and Azores by Morphological and Molecular Characterization
Source: PLoS One. 2012 Apr 19;7(4):e34896. doi: 10.1371/journal.pone.0034896 (PMC3334969; doi:10.1371/journal.pone.0034896)
Supplement: Annex S4 — Measurements (mean values) performed by Delécolle (1985) of four Culicoides species reported for the first time in mainland Portugal. (DOC) [file pone.0034896.s004.doc]

| **Species** | **Wing** | | | **Palp** | | | **Antennae** | | | **Spermathecae** | |
| --- | --- | --- | --- | --- | --- | --- | --- | --- | --- | --- | --- |
|  | **Length (**µ**m)** | **Width (**µ**m)** | **Costa (**µ**m)** | **Length (**µ**m)** | **Ratio 3/(1+2)** | **Third palp segment length (**µ**m)** | **Length (**µ**m)** | **Antennary Index** | **Ratio** | **Number** | **Length (**µ**m)** |
| *C. lupicaris* (♀) | 1787 | 810 | 1142 | 339 | 0,77 | 110 | 870 | 1,11**^*^** | 1,42**^***^** | 2 | First: 86  Second: 72 |
| *C. alazanicus* (♀) | 1285 | 593 | 736 | 202 | 1,35 | 84 | 675 | 1,63**^*^** | 2,26**^***^** | 2 | First: 57  Second: 51 |
| *C. alazanicus* (♂) | 1148 | 406 | 619 | 161 | 1,09 | 52 | 741 | 0,79**^**^** | 2,93**^****^** | n.a. | n.a. |
| *C. deltus* (♀) | 1724 | 776 | 1064 | 279 | 0,84 | 93 | 767 | 1,07**^*^** | 1,46**^***^** | 2 | First: 80  Second: 71 |
| *C. semimaculatus* (♂) | 842 | 357 | 425 | 125 | 0,98 | 40 | 548 | 0,73**^**^** | 3,29**^****^** | n.a. | n.a. |

♀ = Female; ♂ = Male; Costa = Length of the wing from *arculus* to the terminus of second radial cell; Ratio 3/(1+2) = Length of the third palp segment/Length of the first and second palp segments; Antennary Index = *Length of eleventh to fifteenth antennae segments/Length of the third to tenth antennae segments and **Length of thirteenth to fifteenth antennae segments/Length of the third to twelfth antennae segments; Ratio = ***Length of the eleventh antennae segment/Length of the tenth antennae segment and ****Length of the thirteenth antennae segment/Length of the twelfth antennae segment. First spermatheca: mean value of the biggest spermatheca of different specimens; Second spermatheca: mean value of the smallest spermatheca of different specimens; n.a. = Not applicable.
