# Supplementary material for: First Report of 13 Species of Culicoides (Diptera: Ceratopogonidae) in Mainland Portugal and Azores by Morphological and Molecular Characterization
Source: PLoS One. 2012 Apr 19;7(4):e34896. doi: 10.1371/journal.pone.0034896 (PMC3334969; doi:10.1371/journal.pone.0034896)
Supplement: Annex S5 — Measurements (mean values) performed by Delécolle (1985) of four Culicoides species reported for the first time in Azores archipelago. (DOC) [file pone.0034896.s005.doc]

| **Species** | **Wing** | | | **Palp** | | | **Antennae** | | | **Spermathecae** | |
| --- | --- | --- | --- | --- | --- | --- | --- | --- | --- | --- | --- |
|  | **Length (**µ**m)** | **Width (**µ**m)** | **Costa (**µ**m)** | **Length (**µ**m)** | **Ratio 3/(1+2)** | **Third palp segment length (**µ**m)** | **Length (**µ**m)** | **Antennary Index** | **Ratio** | **Number** | **Length (**µ**m)** |
| *C. circumscriptus* (♀) | 1464 | 682 | 832 | 275 | 1,12 | 108 | 721 | 1,35**^*^** | 1,92**^***^** | 1 | 112 |
| *C. circumscriptus* (♂) | 1288 | 501 | 675 | 203 | 1,02 | 67 | 785 | 0,71**^**^** | 2,5**^****^** | n.a. | n.a. |
| *C. newsteadi* (♀) | 1436 | 621 | 820 | 214 | 1,01 | 77 | 631 | 1,01**^*^** | 1,28**^***^** | 2 | First: 66  Second: 64 |
| *C. newsteadi* (♂) | 1172 | 423 | 645 | 184 | 0,98 | 60 | 748 | 0,67**^**^** | 2,23**^****^** | n.a. | n.a. |
| *C. obsoletus sensu stricto* (♀) | 1331 | 640 | 826 | 197 | 0,83 | 61 | 610 | 1,14**^*^** | 1,38**^***^** | 2 | First: 54  Second: 52 |
| *C. obsoletus sensu stricto* (♂) | 1390 | 498 | 827 | 178 | 0,86 | 52 | 715 | 0,78**^**^** | 3,04**^****^** | n.a. | n.a. |
| *C. scoticus* (♀) | 1417 | 679 | 870 | 208 | 0,80 | 64 | 606 | 1,18**^*^** | 1,41**^***^** | 2 | First: 65  Second: 62 |
| *C. scoticus* (♂) | 1497 | 532 | 887 | 176 | 0,83 | 51 | 869 | 0,73**^**^** | 2,97**^****^** | n.a. | n.a. |

♀ = Female; ♂ = Male; Costa = Length of the wing from *arculus* to the terminus of second radial cell; Ratio 3/(1+2) = Length of the third palp segment/Length of the first and second palp segments; Antennary Index = *Length of eleventh to fifteenth antennae segments/Length of the third to tenth antennae segments and **Length of thirteenth to fifteenth antennae segments/Length of the third to twelfth antennae segments; Ratio = ***Length of the eleventh antennae segment/Length of the tenth antennae segment and ****Length of the thirteenth antennae segment/Length of the twelfth antennae segment. First spermatheca: mean value of the biggest spermatheca of different specimens; Second spermatheca: mean value of the smallest spermatheca of different specimens; n.a. = Not applicable.
