# Supplementary material for: First Report of 13 Species of Culicoides (Diptera: Ceratopogonidae) in Mainland Portugal and Azores by Morphological and Molecular Characterization
Source: PLoS One. 2012 Apr 19;7(4):e34896. doi: 10.1371/journal.pone.0034896 (PMC3334969; doi:10.1371/journal.pone.0034896)
Supplement: Annex S6 — Measurements (mean values) performed by Pena (2003) of four Culicoides species reported for the first time in Azores archipelago. (DOC) [file pone.0034896.s006.doc]

| **Species** | **Wing** | | | **Palp** | | | **Antennae** | | | **Spermathecae** | |
| --- | --- | --- | --- | --- | --- | --- | --- | --- | --- | --- | --- |
|  | **Length (**µ**m)** | **Width (**µ**m)** | **Costa (**µ**m)** | **Length (**µ**m)** | **Ratio 3/(1+2)** | **Third palp segment length (**µ**m)** | **Length (**µ**m)** | **Antennary Index** | **Ratio** | **Number** | **Length (**µ**m)** |
| *C. circumscriptus* (♀) | 1350 | 620 | 740 | 234,47 | 1,01 | 87,13 | 671,37 | 1,39 | 1,89 | 1 | 94 |
| *C. newsteadi* (♀) | 1418 | 609 | 807 | 216,27 | 0,95 | 75,6 | 670,8 | 1,03 | 1,31 | 2 | First: 69,5  Second: 62,7 |
| *C. obsoletus sensu stricto* (♀) | 1169 | 553 | 699 | 179,2 | 0,78 | 55,9 | 595,5 | 1,07 | 1,34 | 2 | First: 51,5  Second: 48,6 |
| *C. scoticus* (♀) | 1278 | 596 | 761 | 184,47 | 0,73 | 53,6 | 615,12 | 1,18 | 1,46 | 2 | First: 74  Second: 70,41 |

♀ = Female; ♂ = Male; Costa = Length of the wing from *arculus* to the terminus of second radial cell; Ratio 3/(1+2) = Length of the third palp segment/Length of the first and second palp segments; Antennary Index = Length of eleventh to fifteenth antennae segments/Length of the third to tenth antennae segments; Ratio = Length of the eleventh antennae segment/Length of the tenth antennae segment. First spermatheca: mean value of the biggest spermatheca of different specimens; Second spermatheca: mean value of the smallest spermatheca of different specimens; n.a. = Not applicable.
